# Nutritional Status in Spanish Children and Adolescents with Celiac Disease on a Gluten Free Diet Compared to Non-Celiac Disease Controls

**DOI:** 10.3390/nu11102329

**Published:** 2019-10-01

**Authors:** Catalina Ballestero Fernández, Gregorio Varela-Moreiras, Natalia Úbeda, Elena Alonso-Aperte

**Affiliations:** Department of Pharmaceutical and Health Sciences, Facultad de Farmacia, Universidad San Pablo-CEU, CEU Universities, 28668 Madrid, Spain; cat.ballestero@ceindo.ceu.es (C.B.F.); gvarela@ceu.es (G.V.-M.); nubeda@ceu.es (N.Ú.)

**Keywords:** celiac disease, gluten free diet, nutritional assessment, children, adolescents, dietary intake, nutrient intake, anthropometric measures, physical activity, bone mineral density

## Abstract

Patients who follow a gluten-free diet (GFD) may be prone to nutritional deficiencies, due to food restriction and consumption of gluten-free products. The aim was to assess nutritional status in celiac children and adolescents on a long-term GFD. A cross-sectional age and gender-matched study in 70 celiac and 67 non-celiac volunteers was conducted, using dietary, anthropometric, biochemical parameters, and assessing bone mineral density and physical activity. Adequacy of vitamin D intake to recommendations was very low, in both groups, and intakes for calcium and magnesium were significantly lower in celiac volunteers. Celiac children and adolescents may have a higher risk of iron and folate deficiencies. Both groups followed a high-lipid, high-protein, low fiber diet. Median vitamin D plasma levels fell below reference values, in celiac and non-celiac participants, and were significantly lower in celiac girls. Other biochemical parameters were within normal ranges. Anthropometry and bone mineral density were similar within groups. With the exception of some slightly lower intakes, children and adolescents following a GFD appear to follow the same trends as healthy individuals on a normal diet. No effect of food restriction or gluten-free product consumption was observed.

## 1. Introduction

Celiac disease (CD) is a systemic immune disorder that, after the ingestion of gluten, causes progressive atrophy of the villi in the small intestine of genetically susceptible individuals, resulting in an alteration in the absorption of nutrients, and thus, leading to various deficiency states, although in many cases the disease is asymptomatic [1].

Nowadays, CD is considered a frequent worldwide disease that affects both pediatric and adult patients. Recent epidemiological studies have estimated a CD prevalence of 1:100, with a range between 0.5 to 1.26% [2]. Nonetheless, a significant number of cases remain undetected, with a ratio of 1:3 to 1:5 between diagnosed and undiagnosed cases [3,4]. In Spain, studies conducted in the Community of Madrid, Asturias and The Basque Country provide prevalence data of 1:370 in the adult population and 1:118 to 1:220 in children [5]. Particularly in Madrid, 1 in every 79 children suffers CD [6]. The most frequent clinical presentation of CD is the classical form, mainly diagnosed during the first two years of life [7]. The incidence of CD in Spain has increased notably compared with previous studies, and it is much higher than that observed in other European countries [7].

A strict gluten-free diet (GFD) is the unique treatment for CD: It reduces complications and improves health [8]. The GFD consists of the exclusion of wheat, barley, rye, and all products derived from these cereals, such as starch, flour, breads, pasta, etc.

The patients’ adherence to the GFD diet is the key to successful treatment and prevention of further complications, especially clinical manifestations. A suitable nutritional education program is very important for children, in order to help them willingly accept this diet—taking into account that in many cases the patient may feel deprived of appetizing products, and be tempted by a high consumption of commercial gluten-free highly processed products [9]. These varieties of products are generally high in fat to improve their presentation and palatability [10], and may cause undesirable effects as potential situations of hyperlipemia, overweight or obesity. In fact, a recent review concludes that gluten-free products (GFPs) often have a greater carbohydrate and lipid content than their gluten-containing equivalents, and, on the other hand, they are significantly lower in folates, iron, and overall in B vitamins [11].

Furthermore, it is quite common, just before the diagnosis, to observe a deficiency in some nutrients, such as iron, calcium, zinc, folic acid, vitamin D and other fat-soluble vitamins in patients, as a consequence of malabsorption processes inherent to this pathology. Once patients follow the GFD, the intestinal atrophy is initially restored, allowing the adequate absorption of nutrients [12]. However, some authors have reported an unbalanced diet in terms of macro and micronutrients as a consequence of a bad choice of foods and GFPs [13], but also a potentially lower availability of those foods targeted to the celiac population. Studies undertaken in children and adolescents show that CD patients consume more lipids (especially saturated), protein and simple carbohydrates, but less fiber and micronutrients, such as iron, calcium and vitamin D, than recommended [14,15,16,17,18,19]. In addition, other studies conducted in celiac patients have described an elevated contribution of simple sugars and fat to total energy intake, as compared to healthy subjects [10,20,21]. Nonetheless, some studies have reported a similar total energy intake [21], whilst others higher [10] or lower [20] energy intakes in celiac children as compared to control groups. In general, both groups (healthy and CD children and adolescents), however, failed to accomplish micronutrient recommendations.

CD has traditionally been associated with low weight and height, as well as the presence of lower bone mineral density (BMD). However, more recent studies also indicate that, in children and adolescents, CD may coexist with overweight and obesity [22]. Diamanti et al. [22] show that the prevalence of overweight in CD patients at diagnosis ranges from 8.8% to 20.8%, whereas, that in CD patients on a GFD ranges from 9.4% to 21%. Moreover, the prevalence of obesity in CD patients at diagnosis ranges from 0% to 6%, whereas, in CD patients on a GFD, it ranges from 0% to 8.8% [22]. Normalization of BMD after diagnosis in children with CD is conditioned by strict adherence to a GFD, which is frequently difficult for children. Periodic evaluation of bone status in children, at least until growth detention, seems advisable [23].

For the correct evaluation of children with CD at follow up, a clinical and biochemical evaluation is necessary on a regular basis [24]. However, few studies show data on blood parameters in CD children at follow up. A recent review [25], shows the consequences of a GFD on lipid profile in childhood. In general, at diagnosis CD patients show an altered lipid profile, characterized by elevated total cholesterol and low density proteins (LDL-C), and lower high density lipoprotein cholesterol (HDL-C). A period of GFD increased HDL-C and decreased LDL-C plasma concentrations, but changes in triglyceride and total cholesterol concentrations were different depending on the study analyzed.

Physical activity is not normally evaluated in spite of its importance for BMD and general health status. Babio et al. [21] used a self-reported assessment and found no differences in the level of activity between cases and controls.

The aim of this study was to assess nutritional status in a group of celiac children and adolescents, using dietary, anthropometric and biochemical parameters, as well as assessing bone health and physical activity; in order to identify specific needs and to evaluate possible deficiencies derived from a GFD. To our knowledge, this is the first study in Spanish celiac children and adolescents to undergo a complete nutritional assessment, after they have been on a GFD for at least one year.

## 2. Materials and Methods

### 2.1. Subjects

This is a cross-sectional age and gender-matched study in celiac and non-celiac volunteers. Celiac patients were recruited thanks to the collaboration of the Celiac and Gluten Sensitive Association in Madrid, Spain (*Asociación de Celiacos y Sensibles al Gluten de Madrid*). Participants were children and adolescents diagnosed with celiac disease (CD), both sexes, who belong to the aforementioned Association. Healthy participants (control group), were recruited from the general population. Participants were between 4 and 18 years old. The inclusion criteria for CD patients were: Confirmed medical diagnosis of celiac disease; gluten-free diet (GFD) for over a year; absence of associated diseases; and not taking nutritional supplements. In the case of matched non-celiac controls, inclusion criteria were: Healthy status (absence of diagnosed chronic disease); not having symptoms or signs of any digestive disease; and not taking nutritional supplements. Both groups of participants were excluded if they could not complete the project’s questionnaires properly. Immunoglobulin A (IgA) anti-tissue transglutaminase antibodies (IgA-tTG) were analyzed in blood samples from all participants, in order to identify undiagnosed CD patients, and to screen adherence to the GFD. All values fell within negative values (<6.9 U/mL).

All volunteers (celiac and non-celiac) and legal tutors were informed and provided their written consent to participate in the study. Anonymity was guaranteed. The project was conducted in accordance with legal requirements and guidelines for good clinical practice, as well as the World Medical Association Declaration of Helsinki on Ethical Principles for Medical Research involving Human Subjects (revised in October 2008). The protocol was approved by the Ethics Committee for Human Studies in Universidad San Pablo-CEU (Authorization number 102–15).

### 2.2. Food Habits and Nutrient Intakes

In the first visit, face to face, a dietitian and trained anthropometrist interviewed the participants for personal data, and information about the family history of disease, and medication use. Celiac patients also answered questions about gluten-free products and the easiness to buy and consume them. Anthropometric parameters and bone densitometry, as well as blood sampling, and initial dietary questionnaires were all conducted in this first visit.

For the assessment of the recent diet, we used three 24-h dietary records. The subjects’ usual diet was evaluated by a food frequency questionnaire. In order to estimate food intake, the dietitian conducted the first interview offering help with the use of household portions. Relatives helped the younger volunteers to complete the records. Second and third 24-h dietary records were assessed via phone call with a time difference interval of one month. One of the three 24h records was taken on a Sunday or a holiday. We decided on this methodology following the recommendations by the European Food Safety Authority (Guidance on the EU-Menu methodology) [26].

Volunteers who consumed commercial gluten-free products were asked to record a specific brand. Data on composition, as provided by the manufacture’s label, were recorded, in order to build a composition database on commercial gluten-free products, and use the data for the assessment of nutrient intake. It must be noted, however, that data provided by manufacturers was limited to that under Spanish government regulation; i.e., energy value, amounts of fat, saturated fat, carbohydrates, sugars, protein and salt. Labels do not record data on micronutrient composition, therefore, data on micronutrient intake from these products was not quantified

Data from the three 24-h dietary records were analyzed and compared with the Recommended Intakes of Energy and Nutrients for the Spanish Population [27]. The records were analyzed using the DIAL^®^ software to transform food intake into energy and nutrient consumption. The percentages of adherence to current recommendations were calculated by the following formula: (Observed data/reference data) × 100. Data from the food frequency questionnaires were analyzed to resume the number of meals a day and the frequency of consumption of food by groups (vegetables, fruits, dairy, cereals, cookies and pasta, nuts, etc.). Dietary habits were compared to recommendations [28].

### 2.3. Anthropometric Measures

Anthropometric measurements were taken according to the recommendations of the International Society for the Advancement of Kinanthropometry (ISAK) [29], and by accredited anthropometrists. We measured weight (kg), height (cm), skinfolds of the triceps and subscapular (mm), and circumferences of the waist and arm (cm). We used an electronic scale (Seca 710 scale, Seca gmbh and Co, Hamburg, Germany) for weight, a stadiometer (Seca 213 Telescopic Height Rod for Column Scales, Seca gmbh, Hamburg and Co, Hamburg, Germany) for height, a flexible steel tape (CESCORF, Porto Alegre, Brazil) for the circumferences, and a Harpenden Caliper (The Harpender Skinfold Caliper, Sussex, United Kingdom) for skinfolds.

Body Mass Index (BMI) and percentage of body fat of the participants were calculated according to the weight (kg)/height (m^2^) and Slaughter [30] formulas, respectively. Percentile BMI was compared to the criteria of the World Health Organization (WHO) [31], and Orbegozo Foundation in Spain [32]. Orbegozo defines underweight (<5th percentile), normal weight (5th to < 85th percentile), overweight (85th to < 95th percentile), and obesity (≥95th percentile) in infants. Thus, participants in this study were categorized in subgroups following both criteria. Categorization according to body fat in extreme thinness, thinness, normality, overweight and obesity, following criteria by Marrodán et al. [33] was also assessed.

### 2.4. Blood Parameters

Blood samples were collected in vacutainers, kept at room temperature for 20 min., and then centrifuged at 3000 rpm for 15 min. at 4 °C. The following parameters were determined in plasma: Iron, transaminases (aspartate aminotransferase, AST and alanine aminotransferase, ALT), alkaline phosphatase, phosphorus, folic acid, vitamin D, calcium, growth hormone, glucose, cholesterol, triglycerides, HDL cholesterol, LDL cholesterol (calculated), and Ig A Transglutaminase. LDL cholesterol was calculated according to the formula proposed by Friedewald [34]. All analyses were conducted in a certified laboratory (Megalab, Madrid, Spain).

### 2.5. Bone Mineral Density

The bone mineral density (BMD) analysis was performed by ultrasound bone densitometer HOLOGIC Sahara (Hologic, Marlborough, MA, USA) in the calcaneus. In addition to providing bone mineral density, the ultrasound allows us to measure the mechanical properties of the bone, such as BUA (broadband ultrasound attenuation), which provides data on bone density and trabecular quantity, structure and orientation; and SOS (speed of sound), which indicates the velocity with which the sound can cross the bone, and it depends on the bone’s elasticity and density. This is a non-invasive and practical technique.

### 2.6. Physical Activity

In order to assess physical activity, we used the validated questionnaires PAQ-C (Physical Activity Questionnaire for Children) and PAQ-A (Physical Activity Questionnaire for Adolescents) [35,36]. They include several questions on the physical activity performed in the last seven days. Through these questionnaires, a score in a range of 0 to 5 was assigned, positively valuing the frequency and intensity of each activity, and thus, allowing a quantitative, as well as a qualitative evaluation of physical activity.

### 2.7. Statistical Analysis

Statistical analysis was performed using IBM SPSS^®^ Statistics, version 24.0 (Somers, NY, USA). The variables were checked for normality by the Kolmogorov-Smirnov test, and, in light of the results, the data were expressed as median (percentile 25−percentil 75) for all variables, except for macronutrient distribution and lipid contribution to energy intake, which were expressed as mean ± standard deviation. The comparison between the two groups (celiac and non-celiac), was analyzed for the total sample, and categorized by sex and by age (under 12 years old: Children, and above 12 years old: Adolescents). It was performed using the Mann-Whitney and the Student’s t-tests. Frequencies were expressed as a percentage, and a Pearson’s chi-squared test was used to compare these categorical variables. The level of significance was set at *p* < 0.05 for all analyses.

## 3. Results

The study was finally performed in 70 celiac and 67 non-celiac children and adolescents, aged between 4 and 18 years. Fifty percent of celiac patients were girls. Non-celiac peers were similarly distributed. Eighty percent of celiac patients and 69 percent of non-celiac peers were aged between 4 to 12 years (children), and the rest were adolescents (aged between 13 and 18 years). Results are given for the whole sample and stratified by age and sex.

### 3.1. Food Habits and Nutrient Intake

Volunteers declared taking between 4 to 5 meals a day, usually at home except for school days (0 to 1 meal a week outside the home), and seldom taking fast food (twice a month as the median). There were no significant differences between celiac and non-celiac children and adolescents. All celiac patients declared being on a gluten-free diet for more than a year, and 96.8% stated good adherence. A great part of the sample (84.3%) consumed processed gluten-free products two to three times a day. Only 4.3% of celiac patients avoided gluten-free product consumption. Most celiac participants declared having enough information on celiac disease and product labelling, and they considered themselves capable of choosing proper food products (Table 1). Table 1 shows results on the questions on perception of commercial gluten-free products, expressed as a percentage of positive and negative answers.

Table 2 shows food frequency consumption on a weekly basis. Celiac children and adolescents reported lower consumption of the cereal group (bread, pasta and rice), but no other differences were detected when comparing median portion consumption of other food groups, except for celiac boys, who reported higher consumption of fruits as compared to non-celiac peers. According to meals (data not shown), celiac children and adolescents took a higher amount of milk for breakfast and a lower amount of bread for lunch. In Spain, the midday meal (lunch) provides approximately 40% of total daily energy intake and is, therefore, considered the main meal in the day.

The acceptable macronutrient distribution range recommended for Spanish population proposes that 50 to 60% of total energy should be provided by carbohydrates, 10 to 15% by proteins and up to 35% by lipids [28]. In our study (Table 3), the carbohydrate percentage contribution to energy intake in the diet of celiac children and adolescents was similar to that of non-celiac peers, although it was well below recommendations. The comparison between the two groups showed a significantly lower consumption of proteins in the participants with celiac disease compared to the non-celiac children and adolescents, but intakes meet recommendations. No differences were found for total lipid intake. Protein and lipid contributions to energy intake were high in both groups as compared to recommendations.

In the case of lipids, nutritional objectives for Spanish population propose that saturated fatty acids should not contribute above 7–8% to total energy intake [28]. In our study, both celiac patients and non-celiac children and adolescents consumed saturated fat above recommendations (Table 4). Energy from polyunsaturated fatty acids (PUFAs) and monounsaturated fatty acids (MUFAs) in both groups, however, met nutritional objectives (PUFAs—5% of total energy, MUFAs—up to 20% of total energy [28]. Comparing the two groups studied, there is a significant difference in the contribution of PUFAs to total energy intake, which is lower in volunteers with CD.

Fiber intake is resumed in Table 5. No differences between celiac patients and non-celiac children and adolescents were detected. Dietary fiber intake was low, as compared to recommendations. Nutritional objectives for Spanish population [28], propose a minimum of 22 to 25 g/day of fiber in women and a minimum of 25 to 35 g/day for men.

Finally, Table 6 and Table 7 provide data on mineral and vitamin intake, expressed as a percentage of adherence to current recommendations. We observed a dramatically low intake for vitamin D, reaching scarcely 10 to 15% of recommended intake (15µg/day), in all groups studied (celiac, non-celiac, boys, girls, children and adolescents). Considering a cut-off point of 2/3 recommended intake, intakes may be considered inadequate for folate and vitamin E. Folate intake was significantly lower in celiac patients as compared to non-celiac peers, especially for girls and children. Vitamin E intakes were low in all study groups. Calcium and magnesium intakes were also borderline inadequate, and again celiac patients had lower intakes as compared to non-celiac peers, especially boys and children. Zinc and iodine intakes were borderline adequate, but no differences between celiac and non-celiac children and adolescents were observed. Iron intakes were significantly lower in celiac patients as compared to non-celiac participants, especially for boys and children. Intakes were well above recommended intakes for phosphorus, selenium, thiamin, riboflavin, pyridoxine, vitamin B12, niacin, vitamin C, pantothenic acid, biotin, vitamin A, and vitamin K. When comparing celiac to non-celiac children and adolescents, we found a significantly lower intake for selenium, thiamin, pyridoxine, and niacin, but absolute intakes may be considered adequate.

### 3.2. Anthropometric Measures

Table 8 shows anthropometric characteristics of the total sample, comparing celiac patients and non-celiac children and adolescents, and categorized by sex and age group. No differences were observed in the parameters studied (weight, height, BMI, body fat percentage) between celiac and non-celiac children and adolescents. Over 62% of the sample, both celiac and non-celiac may be classified as normal using body fat percentage cut-off points [33].

Results for bone mineral density (BMD) are resumed in Table 9. Once again, no statistical differences were found due to celiac disease.

**Figure 1:**
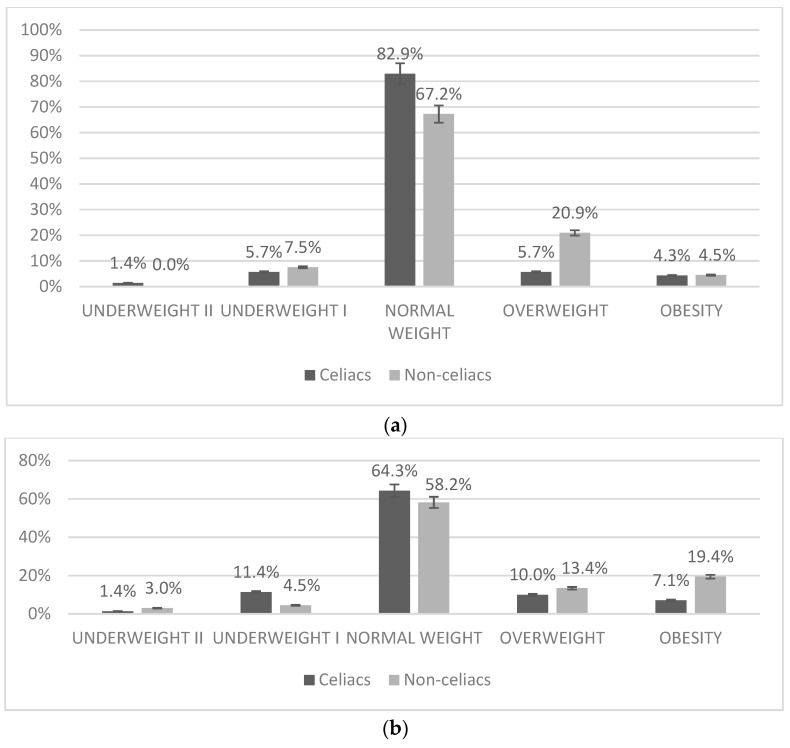
Classification of study participants according to BMI. (**a**) Classification according to BMI as proposed by Orbegozo [32] (**b**) Classification according to BMI as proposed by WHO [31]. Results are expressed as frequency (%) of the total study group.

Participants were classified according to the cut-off points for BMI proposed by *Fundación Orbegozo* (Spain) and the World Health Organization (WHO) (Figure 1). We did not observe statistically significant differences between groups; however, there is a tendency for a higher prevalence of thinness in the group of individuals with celiac disease and a tendency for a higher prevalence of overweight and obesity in the non-celiac children and adolescents.

### 3.3. Blood Parameters

The data obtained in the biochemical study (Table 10 and Table 11) are within the ranges of normality for the parameters studied, except for vitamin D. In the case of vitamin D, values fall under normal reference values (below 30 ng/mL is considered moderate deficiency) in all celiac groups except for boys. When comparing celiac and non-celiac groups, levels were significantly lower in girls. Values for alkaline phosphatase over the range of normality for adults, but within normality for children (reference value < 373 UI/L), in all groups. Iron, folate and calcium blood concentrations were within normality, and no differences between celiac and non-celiac children and adolescents were detected, even though intakes for theses nutrients were low. Glucose and lipid profiles were within normality in all groups.

Comparing the data obtained in the two groups in the biochemical parameters (Table 10 and Table 11), we can observe that there were no significant differences between celiac and non-celiac children and adolescents, except for vitamin D levels and cholesterol relations in girls. When we divided the total sample by gender, we observed a lower level of plasmatic 25-OH vitamin D in the group of celiac girls in comparison to non-celiac girls, being the other way round the boy groups. The difference was statistically significant for girls, but not for boys. In children (ages 4 to 12) and adolescents (ages 13 to 18), we found a moderate deficiency of 25-OH vitamin D in both groups, except for non-celiac girls. To further analyze vitamin D status in relation to celiac disease, we classified participants according to vitamin D plasma concentrations in three groups, and checked for the number of celiac patients within each group. We found no significant relation between celiac disease and vitamin D status (*p* = 0.611, Chi^2^).

Although we found a lower level of plasmatic concentration of 25-OH vitamin D in celiac children and adolescents, and the intake of vitamin D and calcium in the diet was lower than recommendations, we did not find a correlation between plasmatic concentrations of vitamin D and BMD.

### 3.4. Physical Activity

Based on the scores obtained in the PAQ-C and PAQ-A questionnaires, we found no significant differences in the total sample between celiac and non-celiac children and adolescents (Table 12). According to the scores obtained, more than 80% of the celiac subjects fell in a range of moderate to vigorous activity. Most children and adolescents, both study groups, comply with recommendations for physical activity (150 min/week of moderate activity or 75 min/week of vigorous activity) for children and adolescents [37]. The level of physical activity was significantly higher in non-celiac children (4 to 12 years old) and in celiac adolescents (13 to 18 years old).

## 4. Discussion

To the best of our knowledge, this is the first study to asses a complete nutritional status evaluation, using dietary, anthropometric and body composition, biochemical, and physical activity measurements, in Spanish children and adolescents diagnosed with celiac disease (CD), following a gluten-free diet (GFD) for over one year.

Patients who follow a gluten-free diet (GFD) necessarily have to exclude carbohydrate-rich foods containing gluten, and it has been postulated that this restriction may lead subjects with CD to an inadequate choice, and a preference for foods with high caloric fat and protein contents [14,38]. Furthermore, studies show that gluten free commercial products often have greater carbohydrate and lipid content than their gluten counterparts [39,40]. In the present study, we found that 84% of celiac disease children and adolescents eat gluten-free products between two and three times a day. In published studies, CD patients consumed more lipids (especially saturated), protein and simple carbohydrates but less fiber and micronutrients, such as iron, calcium and vitamin D than recommended [14,15,16,17,18,19], and also compared to healthy subjects [10,20,21].

In our study, we did not find relevant differences in the nutrient quality of the diet of children and adolescents following a GFD, as compared to matched controls, in contrast to previous studies. Only folate and polyunsaturated fatty acids intakes were significantly lower in celiac as compared to non-celiac children and adolescents. Nonetheless, micronutrient dietary deficiencies should be further studied before reaching a conclusion, since none of the gluten-free commercial products provides data on micronutrient content. We did, however, observe some inadequacies.

According to the macronutrient distribution profile, both groups followed a high-lipid and high-protein diet compared to the recommendations of the Spanish Society for Community Nutrition [28] for lipids (less than 35% of total energy) and proteins (between 10 and 15% of total energy). Moreover, a low-carbohydrate intake compared to the recommendations (between 50–60% of total energy) of total carbohydrates was found in both groups. This appears to be a general characteristic in the Spanish population in the past few years, as shown by the recent studies ANIBES and ENIDE [41,42]. Significant differences between celiac and non-celiac children and adolescents were found only in the contribution of proteins to total energy intake, with a slightly lower percentage of protein intake in celiac patients as compared to non-celiac peers.

According to the dietary lipid profile, celiac and non-celiac children and adolescents failed to meet the recommendations, with a higher contribution of saturated fatty acids (SFA) to total energy intake, and a lower contribution of mono-unsaturated (MUFAs) and polyunsaturated (PUFAs) fatty acids, as compared to guidelines. Intakes of PUFAs were significantly lower in celiac patients compared to non-celiac subjects. A high intake of SFAs, above dietary recommendations, is common in the European pediatric population [41].

In Spanish infant population, intake of PUFAs varies between 5–17 g per day according to the ANIBES Study [41] so our results show intakes much lower than those of the aforementioned study (2.95 g (0.75–9.00) in non-celiac and 2.40 g (0.56–8.80) in celiac children and adolescents). A possible explanation could be an underestimation due to a lack of information on the PUFA content in the gluten-free products that were consumed by the CD subjects. Nonetheless, non-fortified cereal-based products are not important sources of PUFAs.

Regarding cholesterol intake, in both groups, we found a higher intake than the recommended maximum limit of 300mg/day. These data are similar to those obtained in the ANIBES study [41], which showed a cholesterol intake of 328 mg/day in children and 342 mg/day in adolescents. Intake of trans-type fatty acids is within the acceptable range in the two groups (<3g/day). In the same way, fiber intake recorded in both groups does not accomplish the Recommended Nutritional Objectives for Spanish Population (>25 g per day), being also similar to those obtained in the ANIBES study [41]. These results, altogether, showed an unbalanced diet in terms of macronutrients in both groups, that are in accordance with those shown in several studies [13,14,15,16,17,18,19,20,21]. As regards macronutrients, intakes in celiac children and adolescents are mostly comparable to non-celiac peers.

Studies conducted in different life stages and countries, consistently show that celiac patients are prone to have nutritional complications. At diagnosis, the deficiencies are often secondary to nutrient malabsorption, due to mucosal damage. For CD patients on a GFD, the nutritional complications are likely to be caused by the poor nutritional quality of commercial gluten-free products, and a complicated choice of foods. As revised by Penagini et al. [11], the most common nutrient dietary deficiencies encountered in celiac patients under a GFD are fiber, iron, folate, niacin, vitamin B12 and riboflavin. In our study, we analyzed the contribution of the actual intake to the recommended intakes for these nutrients. In celiac children and adolescents, we did find a significantly lower adequacy for thiamin, riboflavin, pyridoxine, and niacin as compared to non-celiac peers, but intakes in all cases were well above recommendations. Thus, there is no risk of deficiency in these vitamins, at least in our population study group. Vitamin B12 and selenium intakes were also well above recommendations. In the case of iron and folate, celiac patients may have a higher risk for a deficiency, since intakes are significantly lower than in non-celiac children and adolescents, and adequacy to recommended intakes is very close to the cutoff point (2/3 Recommended intake). Nonetheless, data on biochemical parameters were normal for folate and iron plasma concentrations. Therefore, from our data, we can conclude that although celiac patients tend to have a poorer diet in B vitamins and iron, in the absence of anemia or biochemical alterations, these intakes are not prone to generate nutritional complications. Other studies reach the same conclusion, that nutrient intake of children on a GFD diet is mostly comparable to intakes of non-celiac peers, showing the same trends as children on a normal diet [17,18,19].

Other nutrients, such as vitamin D, calcium, and magnesium, may be more problematic for children and adolescents in general, and celiac patients in particular. Adequacy of vitamin D intake to recommendations was dramatically low, both for celiac and non-celiac children and adolescents; and dietary intakes for calcium and magnesium were significantly lower in celiac participants. Zuccotti et al. [10], as well as Óhlund et al. [17] also describe a lower vitamin D intake in CD subjects. Taking into account that the low vitamin D intake was reflected on a moderate vitamin D deficiency as measured in plasma, vitamin D status in Spanish children and adolescents should be further investigated. Interventions with vitamin D-fortified food products for celiac patients could reverse this situation. However, there is a complete lack of nutritional information on vitamins and minerals for gluten-free products of industrial manufacture. Therefore, nutritional assessment using dietary records for celiac patients may be misinterpreted in relation to vitamins and minerals.

Missbach and co-workers [43] have published data on micronutrient content in gluten-free products. They analyzed 63 from originally 162 identified gluten-free foods sold in Austria. They did not analyze the nutritional composition through direct chemical analysis, but estimated the data from the ingredients by deriving data from two nutrient databases. When comparing with gluten-containing counterparts, they found that gluten-free products contained a similar amount of sodium and a lower amount of potassium and zinc, although they did find some differences within food categories. No differences in vitamin content were described. The authors also state that while a majority of gluten-free products are well distributed across European countries, translating their findings to other countries should be interpreted conservatively. Moreover, the presentation of the data (using food categories, but not brands), makes it difficult for us to use their data on our study. We propose that a transnational gluten-free product database should be developed, and other groups working on food composition and celiac disease probably share this idea.

Recent studies have shown that people who follow a GFD may be exposed to a greater accumulation of heavy metals, such as arsenic, mercury, lead, cadmium and cobalt, due to the consumption of foods, such as fish or rice, which are included on GFD. This aspect of the GFD should also be analyzed in subsequent nutritional studies. [44].

The diagnosis of CD has been traditionally related to children and adolescents with low weight and height and/or growth delay, as well as with the presence of lower bone mineral density (BMD) [44]. However, recent studies show that some children with CD are also obese or overweight at diagnosis, a rare, but possible, mode of CD presentation [45].

Studies on anthropometric parameters in children and adolescents with CD following a GFD provide contrasting data: Some studies [11,46] show that the frequency of being overweight increased after one year following a GFD, effect which was also found by Mariani et al. [15] and Norsa et al. [47]. However, other studies show that good compliance with a GFD has a positive effect on body composition [48,49,50] with a normal BMI, in both previously underweight, and overweight subjects. Reilly et al. [51], Bambrilla et al. [52], and Venkatasubramani et al. [53] observed a diminished prevalence of overweight/obesity in patients following a GFD.

The results of the present study (Figure 1) show that, according to cut-off points for BMI proposed by WHO [31], 64.3% of CD subjects and 58.2% of non-celiac children and adolescents had normal weight. The rate of overweight is 10.0% in the celiac group and 13.4% in non-celiac peers. According to WHO criteria, the obesity rate is 7.1% in CD subjects compared to 19.4% of non-celiac children and adolescents, however, these results were not statistically different. Our results, according to the review by Diamanti et al. [22], were within the range found in a majority of published studies. According to Orbegozo Foundation criteria, the prevalence of overweight and obesity, as well as underweight, were lower in CD patients (not statistically significant), and a higher number of subjects were classified as normal weight, as compared to data obtained using WHO criteria. These data are in consonance with the prevalence of obesity and overweight found in recent studies carried out in Spain, such as enKid [54], which found a 24.4% of children and adolescents affected by overweight and obesity. The most recent study in the pediatric population (Aladino, 2015), indicates that the percentage of childhood overweight in Spain is around 23% and that of childhood obesity is around 18% [55].

Our results show a tendency in CD individuals to have a higher prevalence of thinness, as well as a lower presence of obesity, as compared to non-celiac peers. These results coincide with the studies of Brambilla et al. [56] and Van der Pals et al. [57], which found a lower frequency of being overweight and obese in CD children, than in the general population (control group).

With regard to the other anthropometric measures analyzed (triceps fold, subscapular fold, waist circumference, arm circumference, weight, height and body fat percentage), no significant differences were found between the two groups (celiac and controls), even when comparing groups categorized for different age (4 to 12 years, 13 to 18 years) and gender (boys and girls) (data not shown). These results, altogether, suggest that there are no significant differences, from the anthropometric point of view, between celiac children and adolescents on a GFD, and healthy subjects on a normal diet.

The resulting biochemical values were found within normal ranges and not statistically different between groups (Table 9 and Table 10), with two exceptions. Median vitamin D plasma levels fell below reference values for normality (<30 ng/mL), in almost all groups, celiac and non-celiac, bringing forward a moderate vitamin D deficiency. Celiac girls presented a significantly lower level of plasmatic vitamin D as compared to non-celiac controls. Alkaline phosphatase activity, for both groups, was higher than normal reference values for adults, but within normality for children (reference value < 373 UI/L). Alkaline phosphatase activity was significantly lower in celiac adolescents, maybe due to growth. The median age in the celiac adolescents group was 16.0 (15.0–16.0), whilst the median age in the controls was 15.0 (13.0–15.0). Celiac adolescents may have stopped growing, and that would explain lower levels of ALP as compared to the younger group. Levels of physical activity beyond normal ranges, still growing bones, and growth spurts, usually explain the higher levels of alkaline phosphatase in the pediatric and adolescent population [58].

Vitamin D deficiency has been linked, on the one hand, to the risk of suffering autoimmune diseases, and, on the other hand, to the presence of some gastrointestinal disorders or malabsorption syndromes [59]. In theory, a diet based on gluten-free foods would allow the restoration of the intestinal walls and adequate nutrient absorption, and, since no other nutritional deficiencies were found in volunteers with CD, vitamin D deficiency does not seem to be due to a malabsorption syndrome caused by atrophy of the intestinal villi. None of the staple foods that contain vitamin D (i.e., dairy, fish, oils and fat), naturally contains gluten, so the deficit found in children and adolescents with CD would not be attributable to food restriction in the GFD. In addition, intake of this vitamin is insufficient in both groups (celiac and non-celiac), without significant differences between them.

Related to this nutritional deficiency, currently, there is an emerging need for early assessment of bone mineral density (BMD) to treat bone abnormalities in celiac patients, since bone disorders are well documented [60,61,62,63]. Thus, Krzesiek et al. [64] observed a reduction in BMD in 40% of children with diagnosed CD and in 75% in patients with newly diagnosed CD.

In our study, we found no significant differences in the BMD parameters among volunteers with CD and the non-celiac group. Indeed, previous studies [23] show that adherence to the GFD is an important factor to have a minor degree of bone abnormalities, and it can help children and adolescents to recover a normal BMD [65].

The reasons why bone mineralization may be impaired in celiac patients include a decrease in the absorption of the vital nutrients for the bone, such as calcium and vitamin D. In our study, although we found no significant differences in BMD between celiac and non-celiac children and adolescents, we did observe that in subjects with CD, both calcium and vitamin D intakes are well below the recommendations for Spanish Population, and median plasma concentrations of 25-OH vitamin D fell in a moderately deficient range. However, no correlation was found between vitamin D concentration and BMD. Nonetheless, we do advise to assess these parameters in a young population with CD, and to perform an even more selective study of BMD with more specific tests, such as DEXA analysis. Children and adolescents with CD may present problems and difficulties in peaking bone mass achievement, as proposed by other authors [60]. In this same line, some authors suggest adding calcium and vitamin D supplementation to GFDs [66,67,68].

According to the scores obtained for physical activity, more than 80% of the celiac subjects fell in a range of moderate to vigorous activity. Moreover, most children and adolescents, both celiac and non-celiac, complied with the recommendations for physical activity for children and adolescents (150 min/week of moderate activity or 75 min/week of vigorous activity) [37]. A physically active lifestyle is often related to healthy dietary habits so, again, these results on physical activity have probably contributed to a good nutritional status in our study population group.

## 5. Conclusions

Based on our data, children and adolescents with celiac disease following a gluten-free diet for over a year appear to follow the same trends as healthy children on a normal diet, considering the nutrient quality of the diet, anthropometric measures, biochemical biomarkers, bone mineral density, and physical activity. We observed no effect of food restriction or gluten-free product consumption. Special attention should be given to vitamin D, calcium and magnesium intakes, and 25-OH vitamin D plasma concentration should be monitored. When providing information on a gluten-free diet, health professionals and dietitians have a good opportunity to reinforce healthy dietary habits and lifestyles.

The strength of this study is that until now no other study in celiac children and adolescents on a gluten-free diet has carried out a complete nutritional assessment, including: Food habits and dietary analysis, anthropometric and biochemical parameters, bone mineral density assessment, and recording of physical activity. Regarding the weaknesses of the present study, it is important to state that there is a lack of nutritional information about gluten-free products on food composition databases, and labels do not provide information on micronutrient contents. We used all information on labels to assess macronutrient intakes, but vitamin and mineral intakes in celiac children may be, therefore, underestimated.

## Figures and Tables

**Table 1:** Subject perception of commercial gluten-free products in celiac children and adolescents.

Question	Yes
Are you the only one who follows a gluten-free diet at home?	74.3%
Do you have trouble knowing what you can eat and what you cannot?	20.0%
Have you received information on celiac disease and on the labeling of gluten-free products?	84.3%
Would you say that you follow a strict gluten-free diet?	98.6%
Have you noticed improvement in your health since you started on the gluten-free diet?	82.9%
Perceived changes:	
I have lost weight	18.6%
I have gained weight	38.6%
I am more active	42.9%
When you eat away from home, do you have any problem because you suffer celiac disease?	58.6%
Is your diet very different from your siblings/cousins/friends?	25.7%
At home, do you usually buy gluten-free industrially manufactured products?	98.6%
Do you find it difficult to build up a gluten-free menu?	7.1%
You don’t eat more gluten-free products…	
Because you don’t like them	31.4%
Because they are expensive	25.7%
Because you consider that your diet is already complete	51.4%
Your perception of gluten-free products is:	
I find them insipid	18.6%
I find them tasty	44.3%
I like their texture	28.6%
I think they have a similar quality to their gluten-containing counterparts	34.3%
I have never tried products that contain gluten	31.4%

Results are expressed as a percentage.

**Table 2:** Food frequency consumption in celiac and non-celiac children and adolescents, expressed as a number of portions per week.

Food Group	Food Frequency Consumption (Number of Portions/Week)									
	Total Sample		Boys		Girls		Children (4–12 Years)		Adolescents (13–18 Years)	
	Celiac (*n* = 70)	Non-Celiac (*n* = 67)	Celiac (*n* = 35)	Non-Celiac (*n* = 41)	Celiac (*n* = 35)	Non-celiac (*n* = 26)	Celiac (*n* = 56)	Non-Celiac (*n* = 46)	Celiac (*n* = 14)	Non-Celiac (*n* = 21)
Milk and yogurt	12.5 (10.0–14.2)	12.0 (10.0–14.0)	13.0 (10.0–15.0)	11.0 (8.5–14.0)	12.0 (10.0–14.0)	12.0 (10.0–14.0)	12.5 (10.0–14.0)	12.0 (10.0–14.0)	12.0 (9.7–16.2)	12.0 (8.0–14.0)
Fruit	10.0 (6.0–14.0)	8.0 (5.0–12.0)	10.0 * (7.0–15.0)	7.0 (4.0–10.5)	8.0 (6.0–11.0)	10.0 (6.7–13.0)	10.0 (6.0–14.0)	10.0 (6.0–13.0)	7.5 (6.7–11.0)	6.0 (4.0–10.0)
Vegetables	8.0 (4.0–8.0)	8.0 (6.0–8.0)	8.0 (4.0–8.0)	8.0 (6.0–8.0)	7.0 (4.0–8.0)	8.0 (6.0–8.0)	8.0 (4.5–8.0)	8.0 (6.0–8.0)	6.0 (4.0–8.0)	8.0 (6.0–8.0)
Legumes	2.0 (2.0–2.0)	2.0 (2.0–2.0)	2.0 (2.0–2.0)	2.0 (2.0–2.0)	2.0 (2.0–2.0)	2.0 (2.0–3.0)	2.0 (2.0–2.0)	2.0 (2.0–2.0)	2.0 (2.0–2.2)	2.0 (2.0–3.0)
Meat	6.0 (4.0–8.0)	6.0 (4.0–8.0)	6.0 (4.0–8.0)	6.0 (4.5–8.0)	6.0 (4.0–8.0)	6.0 (4.0–8.0)	6.0 (4.0–7.7)	6.0 (4.7–8.0)	6.0 (3.7–8.0)	7.0 (4.0–8.0)
Fish	4.0 (3.0–6.0)	4.0 (3.0–6.0)	4.0 (3.0–6.0)	4.0 (3.0–6.0)	5.0 (3.0–6.0)	3.0 (3.0–6.0)	5.0 (3.0–6.0)	4.0 (3.0–6.0)	3.0 (2.7–5.2)	3.0 (3.0–5.0)
Bread/pasta/rice	14.0 * (11.0–20.0)	18.0 (12.0–22.0)	16.0 (12.0–21.0)	18.0 (13.0–22.5)	14.0 (10.0–19.0)	17.0 (12.0–20.5)	14.0 (10.2–21.7)	18.0 (12.0–22.0)	14.5 * (11.7–16.7)	18.0 (16.0–22.5)
Pastries	6.0 (4.0–8.0)	7.0 (4.0–10.0)	6.0 (3.0–8.0)	7.0 (2.5–10.0)	6.0 (4.0–8.0)	7.0 (4.0–10.0)	6.0 (4.0–8.75)	7.0 (3.5–10.0)	6.0 (3.7–8.0)	6.0 (3.5–9.0)

Results are expressed as median and range (P25–P75). * Significant differences (*p* > 0.05) between celiac and non-celiac participants.

**Table 3:** Macronutrient distribution in the diet of celiac and non-celiac children and adolescents.

Nutrient	Percentage Contribution of Macronutrients Total Energy Intake (%)									
	Total Sample		Boys		Girls		Children (4–12 Years)		Adolescents (13–18 Years)	
	Celiac (*n* = 70)	Non-Celiac (*n* = 67)	Celiac (*n* = 35)	Non-Celiac (*n* = 41)	Celiac (*n* = 35)	Non-Celiac (*n* = 26)	Celiac (*n* = 56)	Non-Celiac (*n* = 46)	Celiac (*n* = 14)	Non-Celiac (*n* = 21)
Carbohydrates	39.9 (35.5–43.5)	39.9 (35.9–4.3)	40.6 (37.2–44.4)	40.1 (35.9–43.7)	38.1 (34.0–42.4)	39.1 (35.0–46.4)	39.8 (35.8–43.7)	39.6 (35.2–43.5)	40.1 (34.2–42.8)	42.4 (37.0–45.6)
Proteins	15.5 * (13.9–16.5)	16.5 (15.3–18.5)	15.2 * (14.2–16.4)	16.4 (15.3–18.7)	16.0 (13.1–16.6)	16.7 (15.3–17.1)	15.3 * (13.9–165–)	16.8 (15.5–18.0)	15.8 (14.5–16.4)	15.7 (14.9–19.9)
Lipids	41.6 (38.1–44.6)	40.7 (37.5–45.6)	40.0 (36.7–43.4)	40.7 (37.8–44.9)	42.4 (38.3–46.7)	41.0 (36.0–46.4)	42.1 (37.3–45.0)	43.2 (37.7–47.3)	40.3 (38.7–41.3)	39.7 (37.0–43.6)

Results are expressed as median and range (P25–P75). * Significant differences (*p* < 0.05) between celiac and non-celiac participants.

**Table 4:** Lipid intake in the diet of celiac and non-celiac children and adolescents.

Nutrient	Percentage Contribution of Lipids to Total Energy Intake (%)									
	Total Sample		Boys		Girls		Children (4–12 Years)		Adolescents (13–18 Years)	
	Celiac (*n* = 70)	Non-Celiac (*n* = 67)	Celiac (*n* = 35)	Non-Celiac (*n* = 41)	Celiac (*n* = 35)	Non-Celiac (*n* = 26)	Celiac (*n* = 56)	Non-Celiac (*n* = 46)	Celiac (*n* = 14)	Non-Celiac (*n* = 21)
PUFA	3.9 * (3.4–4.4)	4.4 (3.8–5.2)	3.7 * (3.5–4.2)	4.3 (2.8–5.2)	4.0 * (3.4–4.5)	4.6 (3.9–5.6)	3.9 * (3.3–4.4)	4.4 (3.7–5.0)	4.2 * (3.5–4.3)	4.6 (4.1–5.6)
MUFA	17.5 (13.9–19.8)	17.9 (14.6–20.7)	15.5 (13.7–18.6)	18.1 (14.5–20.0)	17.9 (14.4–20.1)	17.3 (15.5–21.3)	17.6 (13.9–19.8)	18.7 (15.6–21.3)	17.1 (14.7–19.3)	16.2 (14.0–17.9)
SFA	14.2 (12.8–15.5)	13.8 (12.1–15.6)	13.9 (12.0–14.7)	13.8 (12.1–15.6)	14.5 (12.9–16.0)	13.8 (12.1–15.6)	14.3 (12.2–15.5)	13.9 (12.5–15.9)	14.0 (13.5–14.7)	13.8 (11.51–4.9)

PUFA, polyunsaturated fatty acids; MUFA, monounsaturated fatty acids; SFA, saturated fatty acids. Results are expressed as median and range (P25–P75). * Significant differences (*p* < 0.05) between celiac and non-celiac participants.

**Table 5:** Fiber intake in the diet of celiac and non-celiac children and adolescents.

	Total Sample		Boys		Girls		Children (4–12 Years)		Adolescents (13–18 years)	
	Celiac (*n* = 70)	Non-Celiac (*n* = 67)	Celiac (*n* = 35)	Non-Celiac (*n* = 41)	Celiac (*n* = 35)	Non-Celiac (*n* = 26)	Celiac (*n* = 56)	Non-Celiac (*n* = 46)	Celiac (*n* = 14)	Non-Celiac (*n* = 21)
Fiber (g/day)	16.3 (13.1–20.9)	15.9 (13.6–20.0)	16.5 (15.0–23.2)	15.9 (13.1–19.5)	15.5 (12.4–19.0)	16.1 (13.9–22.0)	16.3 (13.5–20.8)	15.9 (13.7–20.8)	15.7 (12.4–24.3)	15.3 (13.4–17.8)

Results are expressed as median and range (P25–P75).

**Table 6:** Adequacy of mineral intakes in the diet of celiac and non-celiac children and adolescents.

	Percentage Contribution of Mineral Intakes to Recommended Intakes (%)									
	Total Sample		Boys		Girls		Children (4–12 Years)		Adolescents (13–18 Years)	
	Celiac (*n* = 70)	Non-Celiac (*n* = 67)	Celiac (*n* = 35)	Non-Celiac (*n* = 41)	Celiac (*n* = 35)	Non-Celiac (*n* = 26)	Celiac (*n* = 56)	Non-Celiac (*n* = 46)	Celiac (*n* = 14)	Non-Celiac (*n* = 21)
Calcium	64.1 * (24.1–133.2)	74.7 (43.6–145.5)	64.2 * (49.6–70.8)	75.2 (61.9–94.5)	64.5 (52.4–82.9)	74.3 (62.6–93.9)	66.5 * (56.1–77.0)	76.9 (66.5–97.1)	56.9 (45.1–62.4)	65.1 (58.0–78.3)
Phosphorus	115.0 (46.5–265.6)	139.5 (65.6–359.8)	111.9 (97.8–153.9)	140.2 (104–190.6)	125.7 (95.9–191.0)	136.3 (109.2–190.8)	128.2 * (98.5–177.4)	161.4 (115.2–207.5)	101.3 (93.8–112.7)	120.4 (87.6–139.5)
Magnesium	74.4 * (32.4–180.4)	82.8 (29.5–179.4)	62.6 * (54.6–85.8)	78.9 (65.0–94.1)	79.3 (54.3–94.8)	87.6 (72.3–100.7)	76.0 * (57.4–92.9)	88.0 (76.1–103.2)	56.0 (45.2–77.3)	67.0 (62.0–82.8)
Iron	76.0 * (18.9–212.2)	103.1 (46.7–223.3)	76.0 * (64.2–102.5)	110.3 (85.8–133.9)	74.7 (43.9–103.3)	102.2 (59.4–124.4)	81.7 * (55.4–105.0)	116.4 (90.7–150.6)	68.4 (64.2–82.8)	78.3 (61.7–99.2)
Zinc	65.6 (24.0–137.0)	67.5 (32.7–142.0)	66.0 (53.3–82.0)	64.7 (54.7–87.1)	65.4 (43.4–81.0)	69.3 (57.0–89.0)	66.7 * (47.0–83.0)	75.7 (59.0–96.5)	58.7 (46.0–71.1)	56.7 (47.3–69.3)
Iodine	77.8 (27.2–448.9)	79.9 (28.9–170.0)	75.2 (55.9–89.8)	79.2 (72.1–95.2)	92.2 (58.3–107.0)	85.9 (66.3–98.5)	79.7 (57.8–106.6)	86.6 (101.4–76.7)	62.8 (51.5–81.2)	66.3 (52.3–79.3)
Selenium	159.9 * (38.2–416.7)	268.3 (75,0–670,0)	159.9 * (132.4–220.3)	299.2 (181,0–365,0)	155.7 * (126.3–205.1)	234.0 (187.8–313.3)	170.0 * (136.6–223.6)	299.2 (199.3–376.3)	127.6 * (83.3–143.4)	210.0 (177.4–304.7)

Percentage contribution to recommended intakes (%) were calculated by the following formula: (Observed data/reference data) × 100. Results are expressed as median and range (P25–P75). * Significant differences (*p* < 0.05) between celiac and non-celiac participants.

**Table 7:** Adequacy of vitamin intake in the diet of celiac and non-celiac children and adolescents.

	Percentage Contribution of Mineral and Vitamin Intakes to Recommended Intakes (%)									
	Total Sample		Boys		Girls		Children (41–2 Years)		Adolescents (131–8 Years)	
	Celiac (*n* = 70)	Non-Celiac (*n* = 67)	Celiac (*n* = 35)	Non-Celiac (*n* = 41)	Celiac (*n* = 35)	Non-Celiac (*n* = 26)	Celiac (*n* = 56)	Non-Celiac (*n* = 46)	Celiac (*n* = 14)	Non-Celiac (*n* = 21)
Thiamine	110.0 * (36.7–18.2)	133.3 (63.0–300)	110.0 (82.7–45.5)	120.5 (95.0–65.3)	106.1 (91.1–57.1)	149.4 (111.1–77.8)	110.0 * (87.4–58.6)	141.6 (105.2–84.7)	96.4 (82.4–29.7)	120.0 (92.0–56.4)
Riboflavin	107.1 * (37.1–90.0)	121.4 (64.7–25.0)	106.3 (86.9–125.0)	119.7 (93.5–142.5)	107.7 (92.9–150.0)	121.4 (114.3–140.1)	113.3 (93.1–141.7)	129.7 (113.3–150.0)	94.1 (82.0–105.9)	100.1 (88.4–129.4)
Pyridoxine	118.5 * (35.0–312.8)	141.9 (50.6–285.7)	113.4 * (90.5–137.5)	139.3 (99.3–137.5)	120.1 (87.5–136.4)	141.9 (123.8–157.1)	121.4 * (93.8–142.0)	143.2 (125.0–159.8)	95.2 (90.5–102.3)	109.5 (95.2–150.0)
Vitamin B12	276.7 (42.0–1826.7)	295.2 (65.0–1230.0)	276.7 (205.0–338.9)	282.5 (214.2–355.0)	281.7 * (213.3–326.7)	320.0 (248.5–422.8)	270.0 (211.7–320.0)	311.7 (244.3–357.5)	328.1 (204.8–410.0)	263.6 (175.0–422.8)
Niacin	177.4 * (68.2–286.9)	231.4 (76.9–383.9)	185.2 * (154.6–223.4)	227.6 (191.8–270.7)	166.7 (146.2–214.2)	233.3 (196.9–257.6)	184.6 * (155.1–222.7)	237.9 (204.7–276.5)	158.2 (141.8–200.6)	216.5 (158.2–248.4)
Folates	67.45 * (23.3–190.5)	82.0 (32.5–244.5)	61.5 (49.3–89.3)	77.9 (54.8–104.0)	77.5 * (44.8–87.2)	87.7 (56.7–117.2)	77.0 * (48.9–95.0)	89.5 (71.6–117.6)	58.7 (44.2–62.0)	55.0 (47.0–70.3)
Vitamin C	167.9 (19.7–484.0)	151.3 (32.7–447.2)	196.3 (100.7–246.7)	130.6 (88.0–212.5)	153.9 (91.5–232.7)	178.3 (139.8–271.7)	161.7 (94.8–226.8)	169.9 (109.6–245.3)	225.0 (112.2–263.3)	129.7 (85.8–186.7)
Pantothenic acid	118.2 (34.0–180.0)	128.5 (66.0–205.0)	123.5 (104.4–130)	116.8 (108.5–146.7)	119.4 (87.5–152.5)	132.9 (116.0–147.6)	125.0 (100.0–140.0)	135.0 * (115.0–157.5)	106.0 (92.0–116.0)	122.0 (100.0–130.0)
Biotin	143.25 (21.6–414.3)	131.2 (52.5–308.6)	140.5 (97.0–183.6)	125.0 (92.1–169.9)	154.7 (88.4–216.7)	142.5 (96.1–220.1)	158.8 (102.5–214.0)	156.6 (120.3–211.2)	116.0 (88.4–140.9)	91.2 (74.4–131.8)
Vitamin A	97.5 (20.7–343.8)	120.8 (11.7–631.8)	83.7 (57.3–166.8)	100.3 (54.9–185.0)	123.5 (73.1–210.3)	151.2 (100.1–191.3)	110.2 62.6–184.8)	148.5 (95.7–206.4)	77.8 (63.1–119.3)	78.0 (55.0–136.1)
Vitamin D	10.0 (1.2–294.7)	13.2 (0.9–360.0)	11.0 (4.0–18.7)	11.3 (7.4–22.5)	9.0 (6.7–18.0)	15.6 (7.3–26.7)	8.7 (4.8–17.9)	13.6 (6.4–22.4)	14.0 (8.7–22.0)	10.7 (8.0–26.7)
Vitamin E	62.2 (31.0–111.3)	65.0 (20.0–162.5)	62.8 (50.1–77.5)	64.6 (41.9–85.0)	61.0 (40.0–82.0)	70.0 (55.0–88.0)	63.0 (48.6–79.8)	73.1 (58.8–90.0)	55.8 (40.0–72.6)	51.7 (40.0–64.8)
Vitamin K	144.6 (34.9–381.7)	142.8 (30.6–406.7)	145.6 (86.5–196.4)	127.7 (94.5–202.1)	143.0 (74.9–201.8)	148.4 (100.4–169.9)	146.8 (87.8–199.1)	161.5 (105.6–202.1)	93.7 (69.9–197.0)	100.6 (60.7–151.8)

Percentage contribution to recommended intakes (%) were calculated by the following formula: (Observed data/reference data) × 100. Results are expressed as median and range (P25–P75). * Significant differences (*p* < 0.05) between celiac and non-celiac participants.

**Table 8:** Anthropometric characteristics of celiac and non-celiac children and adolescents.

	Total Sample		Boys		Girls		Children (4–12 Years)		Adolescents (13–18 Years)	
	Celiac (*n* = 70)	Non-Celiac (*n* = 67)	Celiac (*n* = 35)	Non-Celiac (*n* = 41)	Celiac (*n* = 35)	Non-Celiac (*n* = 26)	Celiac (*n* = 56)	Non-Celiac (*n* = 46)	Celiac (*n* = 14)	Non-Celiac (*n* = 21)
Weight (kg)	34.1 (23.9–46.9)	35.9 (28.7–51.5)	35.3 (27.5–52.1)	34.3 (28.3–54.3)	33.4 (22.1–45.9)	38.4 (30.4–49.0)	31.6 (22.5–38.3)	31.1 (25.4–37.6)	55.0 (48.1–59.7)	56.4 (49.2–72.7)
Height (cm)	139.9 (123.8–155.4)	141.8 (128.0–159.6)	139.7 (126.4–156.0)	141.8 (127.9–161.0)	140.1 (115.5–155.0)	142.9 (128.2–158.7)	137.5 (119.1–145.9)	134.4 (122.9–142.7)	163.8 (159.1–173.0)	161.4 (158.9–170.7)
BMI (kg/m^2^)	17.0 (15.7–19.2)	18.5 (15.8–21.2)	17.1 (16.0–19.7)	19.1 (16.0–21.9)	16.9 (15.5–18.7)	17.6 (15.7–20.8)	16.7 (15.4–18.3)	17.1 (15.3–19.8)	19.4 (18.3–22.9)	21.6 (19.3–24.8)
Body fat (% of total weight)	16.3 (12.9–22.5)	17.0 (13.5–23.3)	15.2 (11.0–23.3)	14.5 (12.0–22.8)	17.6 (14.1–22.4)	19.1 (15.5–24.9)	16.1 (13.3–22.4)	15.9 (12.4–22.8)	18.2 (10.8–23.3)	21.5 (14.3–24.1)

BMI: Body Mass Index = weight (kg)/height (m^2^). Results are expressed as median and range (P25–P75).

**Table 9:** Bone mineral density in celiac and non-celiac children and adolescents.

	Total Sample		Boys		Girls		Children (4–12 Years)		Adolescents (13–18 Years)	
	Celiac (*n* = 70)	Non-Celiac (*n* = 67)	Celiac (*n* = 35)	Non-Celiac (*n* = 41)	Celiac (*n* = 35)	Non-Celiac (*n* = 26)	Celiac (*n* = 56)	Non-Celiac (*n* = 46)	Celiac (*n* = 14)	Non-Celiac (*n* = 21)
BMD (g/cm^2^)	0.522 (0.467–0.593)	0.517 (0.464–0.576)	0.540 (0.468–0.565)	0.520 (0.466–0.568)	0.515 (0.454–0.575)	0.505 (0.459–0.614)	0.517 (0.458–0.579)	0.514 (0.464–0.552)	0.602 (0.501–0.720)	0.517 (0.459–0.633)

Results are expressed as median and range (P25–P75).

**Table 10:** Biochemical parameters in celiac and non-celiac children and adolescents.

	Total Sample		Boys		Girls		Children (4–12 Years)		Adolescents (13–18 Years)	
	Celiac (*n* = 67)	Non-Celiac (*n* = 66)	Celiac (*n* = 33)	Non-Celiac (*n* = 41)	Celiac (*n* = 34)	Non-Celiac (*n* = 25)	Celiac (*n* = 53)	Non-Celiac (*n* = 45)	Celiac (*n* = 14)	Non-Celiac (*n* = 21)
Iron (µg/dL)	98.0 (75.0–123.0)	102.5 (83.0–123.0)	90.0 (74.0–109.0)	101.0 (83.0–116.0)	108.0 (84.0–132.0)	109.0 (83.0–123.0)	98.0 (79.0–123.0)	104.0 (84.0–124.0)	95.5 (68.0–122.0)	101.0 (82.0–110.0)
AST (U/L)	30.0 (26.0–35.5)	31.0 (26.0–37,0)	31.0 (27.0–37.0)	31.0 (27.0–37.0)	28.5 (24.0–33.0)	28.0 (25.0–33.0)	31.0 (27.0–36.0)	32.0 (28.0–38.0)	26.0 (19.0–32.0)	25.0 (21.0–31.0)
ALT (U/L)	27.0 (23.0–31.0)	29.0 (24.0–32.0)	28.0 (24.0–32.0)	30.0 (26.0–32.0)	26.0 (22.0–31.0)	27.0 (23.0–30.0)	27.0 (23.0–31.0)	29.0 (25.0–33.0)	29.0 (27.0–40.0)	27.0 (22.5–30.0)
Alkaline Phosphatase (U/L)	197.0 (161.5–228.5)	207.0 (171.0–251.0)	195.0 (162.0–239.0)	207.0 (182.0–266.0)	198.0 (161.0–225.0)	202.0 (142.0–249.0)	210.0 (188.0–237.0)	211.0 (193.0–274.0)	78.5 (54.0–159.0)	141.0 (89.0–222.0)
Phosphorus (mg/dL)	5.0 (4.7–5.4)	5.0 (4.7–5.4)	5.1 (4.7–5.4)	5.0 (4.7–5.4)	5.0 (4.7–5.3)	5.0 (4.6–5.3)	5.1 (4.9–5.5)	5.1 (4.9–5.5)	4.4 (4.2–5.0)	4.7 (4.2–5.0)
Folate (ng/mL)	6.7 (4.3–9.1)	6.0 (4.5–8.2)	7.1 (4.3–9.9)	6.0 (4.2–8.5)	6.4 (4.3–8.4)	6.0 (4.8–7.5)	7.0 (4.4–9.9)	6.9 (4.6–10.8)	5.0 (4.1–7.2)	4.9 (3.6–6.0)
Vitamin D (ng/mL)	28.0 (22.8–33.9)	28.9 (23.9–35.8)	30.6(4.3–36.5)	26.6 (23.7–33.7)	25.9 * (21.9–32.6)	30.7 (25.8–36.5)	27.9 (22.9–33.6)	29.8 (24.1–36.5)	28.8 (22.7–35.8)	26.1 (23.0–32.3)
Calcium (mg/dL)	9.8 (9.6–10.0)	9.8 (9.6–9.9)	9.8(9.7–10.0)	9.8 (9.5–10.0)	9.8 (9.7–10.0)	9.7 (9.5–10.0)	9.8 (9.6–10.0)	9.8 (9.6–10.0)	9.7 (9.6–10.0)	9.7 (9.5–9.9)
Growth hormone (ng/mL)	0.3 (0.1-3.5)	0.4 (0.1–1.9)	0.5 (0.3–1.0)	0.2 (0.1–0.2)	1.9 (0.2–5.3)	0.5 (0.3–2.5)	0.3 (0.1–3.1)	0.4 (0.1–1.4)	1.0 (0.3–7.3)	0.4 (0.1–2.5)

Results are expressed as median and range (P25–P75). * Significant differences (*p* < 0.05) between celiac and non-celiac participants.

**Table 11:** Biochemical parameters in celiac and non-celiac children and adolescents.

	Total Sample		Boys		Girls		Children (4–12 Years)		Adolescents (13–18 Years)	
	Celiac (*n* = 46)	Non-Celiac (*n* = 48)	Celiac (*n* = 22)	Non-Celiac (*n* = 34)	Celiac (*n* = 24)	Non-Celiac (*n* = 14)	Celiac (*n* = 36)	Non-Celiac (*n* = 34)	Celiac (*n* = 10)	Non-Celiac (*n* = 14)
Glucose (mg/dL)	79.5 (76.0–84.0)	79.5 (76.0–83.5)	79.5 (76.0–84.0)	80.0 (76.0–85.0)	80.0 (77.0–83.5)	78.5 (73.7–82.0)	80.5 (76.0–83.5)	80.0 (76.0–84.0)	79.0 (69.0–84.0)	79.0 (78.0–82.0)
Cholesterol (mg/dL)	167.0 (143.0–184.0)	163.5 (152.0–182.0)	168.0 (146.0–177.0)	165.0 (152.0–189.0)	165.5 (142.5–194.0)	162.5 (153.0–182.2)	168.5 (142.5–186.5)	165.0 (154.0–190.0)	159.0 (150.0–184.0)	158.5 (140.0–179.0)
Triglyceri-des (mg/dL)	42.5 (38.0–59.0)	52.0 (38.0–62.5)	42.5 (35.0–61.0)	52.0 (38.0–60.0)	40.5 (35.7–61.5)	52.0 (37.2–61.5)	42.0 (37.5–56.5)	52.0 (38.0–62.0)	55.5 (38.0–61.0)	50.0 (38.0–60.0)
HDL-Cholesterol (mg/dL)	64.5 (54.0–74.0)	64.5 (59.0–75.5)	70.5 (54.0–76.0)	63.0 (59.0–71.0)	64.0 (55.0–69.5)	66.5 (60.0–81.0)	64.0 (55.0–72.0)	68.5 (59.0–77.0)	70.5 (54.0–76.0)	60.0 (59.0–67.0)
LDL-Cholesterol (mg/dL)	92.1 (73.2–100.4)	88.7 (76.9–101.6)	86.9 (72.0–98.6)	89.1 (75.2–18.6)	94.3 (78.8–117.2)	86.8 (78.8–92.6)	94.1 (76.1–100.6)	89.2 (83.2–99.0)	80.8 (72.0–97.8)	78.8 (72.5–109.7)
Total Cholesterol/HDL Cholesterol	2.4 (2.3–2.7)	2.4 (2.2–2.9)	2.4 (2.2–2.6)	2.6 (2.2–3.1)	2.6 * (2.4–2.9)	2.3 (2.2–2.7)	2.5 (2.2–2.9)	2.4 (2.2–2.9)	2.5 (2.2–2.6)	2.5 (2.0–3.0)
LDL-Cholesterol /HDL-Cholesterol	1.3 (1.2–1.6)	1.3 (1.1–1.8)	1.4 (1.1–1.8)	1.3 (1.1–1.4)	1.4 * (1.3–1.7)	1.2 (1.1–1.5)	1.4 (1.2–1.7)	1.3 (1.1–1.8)	1.3 (1.0–1.4)	1.4 (0.9–1.8)

Results are expressed as median and range (P25–P75). * Significant differences (*p* < 0.05) between celiac and non-celiac participants.

**Table 12:** Physical activity scores for celiac and non-celiac children and adolescents.

	Total Sample		Boys		Girls		Children (4–12 Years)		Adolescents (13–18 Years)	
	Celiac (*n* = 70)	Non-celiac (*n* = 67)	Celiac (*n* = 35)	Non-Celiac (*n* = 41)	Celiac (*n* = 35)	Non-Celiac (*n* = 26)	Celiac (*n* = 56)	Non-Celiac (*n* = 46)	Celiac (*n* = 14)	Non-Celiac (*n* = 21)
Score	2.5 (2.0–3.2)	2.7 (2.2–3.3)	2.5 (2.0–3.2)	2.8 (2.2–3.5)	2.5 (1.9–3.2)	2.6 (2.3–3.1)	2.5 * (2.0–3.2)	3.0 (2.5–3.4)	2.8 * (2.2–3.1)	2.2 (1.7–2.7)

Results are expressed as median and range (P25–P75).
